# An Improved Genome Assembly for *Drosophila navojoa,* the Basal Species in the *mojavensis* Cluster

**DOI:** 10.1093/jhered/esy059

**Published:** 2018-11-13

**Authors:** Thyago Vanderlinde, Eduardo Guimarães Dupim, Nestor O Nazario-Yepiz, Antonio Bernardo Carvalho

**Affiliations:** 1 Departamento de Genética, Instituto de Biologia, Universidade Federal do Rio de Janeiro, Rio de Janeiro, Brazil; 2 Laboratorio Nacional de la Genómica para la Biodiversidad, Centro de Investigación y Estudios Avanzados del Instituto Politécnico Nacional (CINVESTAV), Irapuato, Guanajuato, México

**Keywords:** Genomics and gene mapping, Bioinformatics and computational genetics, cactus, *Drosophila navojoa*, genome assembly, *mojavensis* cluster, *repleta* group, transcriptome

## Abstract

Three North American cactophilic *Drosophila* species, *D. mojavensis*, *D. arizonae*, and *D. navojoa,* are of considerable evolutionary interest owing to the shift from breeding in *Opuntia* cacti to columnar species. The 3 species form the “*mojavensis* cluster” of *Drosophila*. The genome of *D. mojavensis* was sequenced in 2007 and the genomes of *D. navojoa* and *D. arizonae* were sequenced together in 2016 using the same technology (Illumina) and assembly software (AllPaths-LG). Yet, unfortunately, the *D. navojoa* genome was considerably more fragmented and incomplete than its sister species, rendering it less useful for evolutionary genetic studies. The *D. navojoa* read dataset does not fully meet the strict insert size required by the assembler used (AllPaths-LG) and this incompatibility might explain its assembly problems. Accordingly, when we re-assembled the genome of *D. navojoa* with the SPAdes assembler, which does not have the strict AllPaths-LG requirements, we obtained a substantial improvement in all quality indicators such as N50 (from 84 kb to 389 kb) and BUSCO coverage (from 77% to 97%). Here we share a new, improved reference assembly for *D. navojoa* genome, along with a RNAseq transcriptome. Given the basal relationship of the *Opuntia* breeding *D. navojoa* to the columnar breeding *D. arizonae* and *D. mojavensis*, the improved assembly and annotation will allow researchers to address a range of questions associated with the genomics of host shifts, chromosomal rearrangements and speciation in this group.

The *repleta* group is one of the largest species radiations in the *Drosophila* genus. It contains at least 100 species (Markow and O’ Grady 2005), and most of them breed on fermenting cactus tissues in semiarid or arid environments (Ruiz and Heed 1988; Markow and O’ Grady 2005). These species originated in North, Central, and South America, and represent an important model system for studies in ecology, genetics, and speciation. Their evolutionary relationships are well-defined (Wasserman 1982; Durando et al. 2000; Oliveira et al. 2012) and the group is characterized by a larger number of chromosomal inversions than observed in other *Drosophila* lineages (González et al. 2007).

The first *repleta* species sequenced was *Drosophila mojavensis* (Drosophila 12 Genomes Consortium 2007). *Drosophila mojavensis* and its sister species, *Drosophila arizonae*, and *Drosophila navojoa*, form a triad known as the *mojavensis* cluster (Ruiz et al. 1990). They are closely related (Figure 1), but have important differences in their chromosome inversions (Ruiz et al. 1990) and ecology (Heed 1978; Reed et al. 2007; Pfeiler et al. 2009). *Drosophila navojoa* is the more basal and its distribution is restricted to the west coast of Mexico’s mainland, where it breeds exclusively in *Opuntia* cactus. *Drosophila mojavensis* is more widespread than *D. navojoa*, occurring in southern California, Arizona, Sonora, Sinaloa, and the Baja California peninsula, where it primarily utilizes various columnar cactus species (Ruiz and Heed 1988). The most geographically widespread of them is *D. arizonae*, reported from Guatemala to southern United States. While both *D. mojavensis* and *D. arizonae* utilize columnar cacti as hosts, both species may utilize *Opuntia* in parts of their ranges.

**Figure 1.**
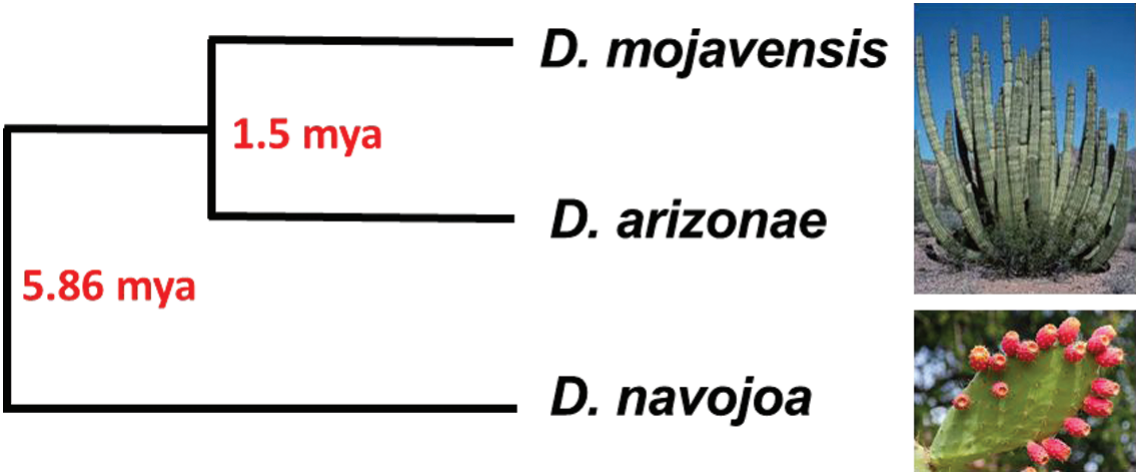
Evolutionary relationships and host cactus use in the ancestral *Drosophila navojoa* and the derived *Drosophila mojavensis* and *Drosophila arizonae*, member of the *mojavensis* cluster. Divergence times were taken from Sanchez-Flores et al. 2016.

Recently the *D. navojoa* and *D. arizonae* genomes were sequenced (Sanchez-Flores et al. 2016), opening many avenues to study the genetic basis of ecological divergence, chromosome structure, and speciation. Both genomes were sequenced and assembled by the same team using the same methods: DNA was extracted from adult males of inbred lines, sequenced using Illumina and the genomes were assembled *de novo* with AllPaths-LG software (Ribeiro et al. 2012). Therefore, both genomes were expected to have similar quality, although the amount and type of repetitive sequences, which cause the majority of assembly problems, can vary even between closely relates species (Jagannathan et al. 2017). These differences might explain the finding that the *D. navojoa* assembly was more fragmented, incomplete, and thus less useful than the genome of *D. arizonae*, as reported by Sanchez-Flores et al. (2016).

Given the importance of *D. navojoa*, we tried an alternative assembly approach with the same reads, and indeed obtained a significantly improved assembly of *D. navojoa*, which is now similar in quality to that of *D. arizonae*. The *D. navojoa* genome was originally assembled using AllPaths-LG, which is an efficient assembler tested with many organisms (Gnerre et al. 2011). This software requires “super-reads” which are assembled from a 100 bp paired-end library with an average insert size of 180 bp. Note that in such libraries the forward and reverse reads of each insert would have an overlap of ~20 bp, which is used to convert each forward/reverse pair into a ~180 bp single-end read (called a “super-read”). After this initial step, the genome itself was assembled using the super-reads (Ribeiro et al. 2012). However, the insert size of the main *D. navojoa* library seems too large for the efficient assembly of super-reads (average: 257 bp; Table 1 in Sanchez-Flores et al. 2016). The *D. arizonae* library, on the other hand, had an insert size which, although a bit small (145 bp), would have allowed the efficient assembly of super-reads. Hence, it occurred to us that poor super-read assembly might be the cause of *D. navojoa* assembly problems.

**Table 1.** Assembly statistics for *Drosophila navojoa* from the original (AllPaths-LG) and new (SPAdes) assemblies compared to that of *Drosophila arizonae*

Assembly statistics	*D. navojoa*		*D. arizonae*
	Original assembly	New assembly	Original assembly
N50	82455	389283	171766
Total number of scaffolds	10779	13813	5133
Sum (Mbp)	115	147	141
Maximum scaffold size (bp)	1117492	3635071	1311587
Complete BUSCOs (%)	76.7	97.4	93.4
Complete and single-copy BUSCOs (%)	76.4	97	92.9
Complete and duplicated BUSCOs (%)	0.3	0.4	0.5
Fragmented BUSCOs (%)	4.8	1.4	2.1
Missing BUSCOs (%)	18.5	1.2	4.5

Another aspect in which *D. navojoa* lags behind its sister species is in gene expression data, which currently is available only for *D. mojavensis* and *D. arizonae*, and their hybrids (Wagstaff and Begun 2005; Matzkin 2012; Lopez-Maestre et al. 2017; Nazario-Yepiz et al. 2017). We remedied this situation, by performing RNAseq in multiple life stages (adults, pupae, and larvae), which resulted in an improved gene annotation.

## Material and Methods

### *De novo* Genome Assembly

If the problem in the insert size in the paired-end library was real, *D. navojoa* (but not *D. arizonae*) should have a small percentage of overlaps between forward and reverse reads. In order to verify this, Flash (Magoč and Salzberg 2011), a software that finds overlaps between forward and reverse paired-end reads, was applied to both *D. navojoa* and *D. arizonae* reads. The parameters used for Flash, as for all the programs used in this section, are available in the Supplementary Material.

In order to test the hypothesis of poor super-read assembly, and to try to improve the *D. navojoa* genome, we re-assembled the same paired-end and mate-pair libraries with the SPAdes software (Bankevich et al. 2012) which is not based on super-reads. SPAdes was originally designed for assembly of single-cell bacterial genomes, and it has been shown to produce very good assemblies of a protist (Seddiki et al. 2018), fungi (Abbas et al. 2014), nematodes (Yin et al. 2018), and insects (Prokhortchouk et al. 2017).

After the initial AllPaths-LG assembly, Sanchez-Flores et al. (2016) used the *D. mojavensis* genome to group and orient the *D. navojoa* scaffolds that presumably belong to same Muller element, using the software ABACAS (Assefa et al. 2009). This procedure greatly reduces the number of scaffolds and leads to a chromosome-level assembly. This is achieved somewhat artificially, however, by assuming conservation of gene content and order in the chromosome arms. We report here the primary SPAdes assembly and compared it with the primary AllPaths-LG assembly produced before.

### Removal of Bacterial Contaminants

The DNA from adult flies is expected to contain some contaminants (e.g., microorganisms from digestive tract). We used the software Blobtools (Laetsch and Blaxter 2017) to identify them, for posterior removal. Blobtools uses 3 pieces of information to identify contaminants: 1) the concentration of contaminant DNA usually is small in comparison with the fly DNA, and hence in the final assembly the contaminant sequences will have a smaller coverage; 2) many bacterial genomes are more GC-rich when compared to eukaryotes; and 3) a BLASTX search against RefSeq proteins database (downloaded from ftp://ftp.ncbi.nlm.nih.gov/blast/db/).

### RNA Sequencing and Transcriptome *de novo* Assembly

The same strain of *D. navojoa*, from Jalisco, México, that was used for sequencing the genome (Sanchez-Flores et al. 2016) was used for the RNA sequencing. We used multiple life stages of *D. navojoa*: 30 third instar larvae, 30 pupae, 30 adult females, and 30 adult males.

All samples were washed twice with cold distilled water, placed into 1.5-ml tubes, and then rinsed twice with 1X PBS; all liquid was removed, and the material was used for RNA extraction using the Direct-zol RNA MiniPrep kit (Zymo Research) according to the manufacturer’s protocol. Briefly, the samples were homogenized with TRI-Reagent using Teflon homogenizers, and the RNA was purified using columns. Three aliquots of each sample were saved, one to measure RNA concentration by NanoDrop (Thermo Scientific), another for analyses in a 1% agarose gel, and one for the sequencing core facility at LANGEBIO.

Libraries were prepared with TruSeq RNA Sample Preparation Kit v2 (Illumina), selecting only polyA mRNAs and synthesizing double-stranded cDNAs to attach to the Illumina adapters. Library size and quality were measured by Bioanalyzer (Agilent Technologies) and sequenced in a 2 × 300 paired-end read format on a MiSeq Sequencing System (Illumina).

The transcriptome was assembled using 2 different assemblers, in order to indentify the best draft transcriptome. We ran Trinity (Amit et al. 2013) and rnaSPAdes (Bankevich et al. 2012) with default parameters, pooling the reads from the 4 libraries.

### Completeness of Genome and Transcriptome Assemblies

The completeness of genome and transcriptome assemblies were assessed using the software BUSCO (Simão et al. 2015), which measures the proportion of highly conserved Diptera genes present in the assemblies. Sanchez-Flores et al. (2016) used CEGMA (Parra et al. 2007) for the same purpose; unfortunately it was discontinued (http://www.acgt.me/blog/2015/5/18/goodbye-cegma-hello-busco) and therefore we used BUSCO in all analysis. We ran BUSCO with the OrthoDb v9 set of Diptera (diptera_odb9).

To better illustrate the genome assembly improvement, we performed TBLASTN (Altschul et al. 1990) using as queries a random sample of 10 genes, chosen without any prior information on their completeness in the assemblies (they are commonly used in phylogenetic studies). The genes are *patched*, *even skipped, ebony*, *engrailed*, *Dopa decarboxylase*, *Notum*, *wingless*, *hedgehog*, *Distal-less*, and *Amyrel*. In all cases, we used the *D. melanogaster* protein sequences as the query in the TBLASTN searches.

### Gene Prediction and Functional Annotation

A new genome assembly becomes more useful when complemented with a new annotation. This was done with Augustus (Stanke et al. 2006) using optimization training for the *D. navojoa* made by BUSCO (--long option). Additionally, we used the new transcriptome as evidence for the prediction (--hintsfile), following the instructions of Augustus documentation (“6. Predictions using cDNA”, in README.txt). Similarly to the raw genome, the completeness of the gene prediction set was analyzed by BUSCO (--mode protein). The predicted proteins were annotated as described by Sanchez-Flores et al. (2016). Briefly, the proteins from *D. navajoa* gene models are compared with proteins from *Drosophila* species from FlyBase.org, and clustered using CD-HIT v.4.6 (Li and Godzik 2006), with a cutoff value of 80% identity using default parameters. The Uniprot ID and short name for each protein were obtained by matching the FlyBase IDs in the clusters with custom shell and Perl scripts and relational files obtained from Uniprot (ftp://ftp.uniprot.org/pub/databases/uniprot/current_release/knowledgebase/complete/docs/fly.txt and ftp://ftp.uniprot.org/pub/databases/uniprot/current_release/knowledgebase/complete/docs/shortdes.txt).

## Results

### The Improved Genome Assembly

Given the estimated insert size of the *D. navojoa* main library (average of 257 bp; Sanchez-Flores et al. 2016), it is expected that in many read pairs it will not be possible to combine the Forward and Reverse reads into a single super-read. Indeed, the Flash software could only produce super-reads in 3.7% of the read pairs, whereas in the *D. arizonae* dataset we got a yield of 62.5%. Hence, the excessively large insert size of the *D. navajoa* main library was detrimental to the formation of super-reads. It might be surprising that the original *D. navojoa* assembly succeeded, given the low yield of super-reads. As a tentative explanation, note that the *D. navojoa* genome was sequenced at a high depth (81-fold; Table 1 of Sanchez-Flores et al. 2016), and hence the 3.7% yield of super-reads amounts to 3-fold coverage, which is very low, but probably enough for the initial steps of the AllPaths-LG software.

Our initial SPAdes assembly contained 14376 scaffolds (total size of ~147.8 Mbp). Guided by the plot generated by Blobtools (Supplementary Figure 1), we removed all 563 scaffolds with coverage below 4× (largest scaffold size = 4426 bp, total size ~0.4 Mbp). All these low coverage scaffolds seem to be contaminants, mostly from Proteobacteria. The final assembly has 13813 scaffolds (total size of 147.3 Mbp).

**Figure 2.**
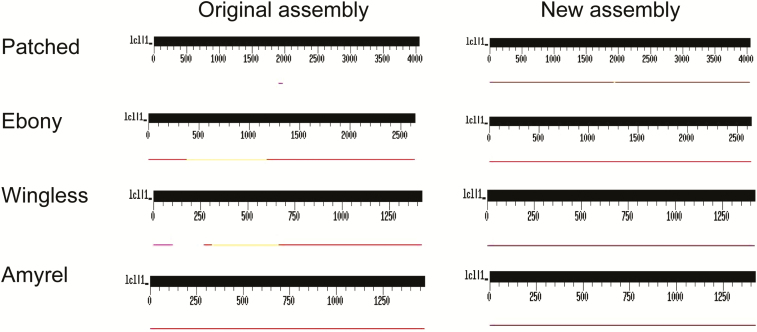
Completeness of a random sample of genes in the original and in the improved assemblies of *Drosophila navojoa*. The genes were chosen because they are commonly used in phylogenetic studies, without any prior information of their completeness in both assemblies. Seven of them are complete in both assemblies (*Amyrel*, *even skipped*, *engrailed*, *Dopa decarboxylase*, *Notum*, *hedgehog*, and *Distal-less*; we represented only the first one). The remaining 3 genes are complete in the new assembly but are missing parts (or are altogether absent) in the original assembly (*patched*, *ebony* and *wingless*). In all cases we used the protein sequence of *Drosophila melanogaster* ortholog as the query in a TBLASTN search.

Table 1 compares the new *D. navojoa* assembly with the previous one and that of *D. arizonae*. All assembly quality indicators demonstrate major improvements: there was a huge increase in N50 (4-fold), maximum scaffold size (3-fold), total sequence assembled, etc. In addition, fewer conserved genes are now missing or fragmented from new assembly, as assessed by BUSCO. A perhaps more intuitive view of the improvement is shown in Fig. 2, in which we performed a TBLASTN search, using as queries a random sample of 10 genes. Again, the new assembly clearly is better.

### Transcriptome for *D. navojoa*

We report here the first transcriptome for *D. navojoa*, which covers different life stages (larvae, pupae, adult males, and adult females) Using Trinity we obtained a total of 69635 transcripts, whereas SPAdes assembled 22589 sequences. We then ran BUSCO, in order to assess the completeness of these transcriptomes and help to choose which one will be used as evidence in the gene prediction (Table 2). The completeness was very similar (2% difference), but the Trinity assembly contains a much larger number of duplicated genes. Given that the assembled *D. navojoa* genome does not have a large number of duplicated genes (Table 1), those observed in the Trinity assembly probably are artifacts. Thus, we used the rnaSPAdes assembly as the first draft transcriptome for *D. navojoa*.

**Table 2.** Assembly statistics for *Drosophila navojoa* transcriptome

Assembly statistics	Trinity	rnaSPAdes
Total number of scaffolds	69635	22589
Complete BUSCOs (%)	89	87
Complete and single-copy BUSCOs (%)	50	81
Complete and duplicated BUSCOs (%)	39	6
Fragmented BUSCOs (%)	8	7
Missing BUSCOs (%)	3	6

### Gene Prediction and Annotation

As expected, the assembly improvement was also reflected in the gene annotation. The new assembly has 15596 protein-coding genes (97% of complete BUSCOs, 1% missing BUSCOs), while the previous assembly deposited in NCBI (assembly number ASM165401v1) has 15855 genes (79% of complete BUSCOs, 17% missing BUSCOs). The number of predicted genes seems inflated in comparison to the *D. mojavensis* reference genome (15015 genes), possibly because of the fragmentation of the assemblies, but the BUSCO results show that the new annotation has fewer missing genes and a higher completeness. Taking into consideration both the genome and the annotation statistics, we believe that there is strong justification to use the new assembly as the standard *D. navojoa* assembly. It seems likely that the primary problem of the previous assembly was an incompatibility between the average insert size of the main Illumina library (257 bp) and the requirements of AllPaths-LG.

## Discussion

The sequencing of the *D. navojoa* genome resulted in a reasonably good draft assembly (Sanchez-Flores et al. 2016) and the new assembly using SPAdes has improved its contiguity and completeness. The hypothesis that the inadequate insert size had an adverse effect on the performance of AllPaths-LG in the previous assembly seems likely. Genome sequencing is quickly becoming a standard tool for many types of biological studies, ranging from mutation identification (Yin et al. 2018) to speciation and phylogenetic studies (Sanchez-Flores et al. 2016) and classification (Das and Hirano 2012). Differences in assembly quality can negatively impact all these applications (Khan et al. 2018), and hence improved assemblies are always desirable.

The availability of the genomes of *D. mojavensis*, *D. arizonae*, and *D. navojoa* opens a wide range of investigations into the processes of ecological adaptation and speciation (Sanchez-Flores et al. 2016). An improved assembly and annotation of the *D. navojoa* genome will facilitate these studies. For example, gene expression profiles following experimental host plant shifts in *D. mojavensis* revealed the importance of detoxification genes in this process (Matzkin et al. 2006; Matzkin 2012). In those experiments, *D. mojavensis* was reared on different species of closely related columnar cacti. The shift between *Opuntia* and columnars has not been addressed previously for lack of a better *D. navojoa* genome. A critical step, therefore, given that the switch from *Opuntia* to columnar cacti has occurred more than once in the *repleta* species group, will be to examine the expression profiles of *D. navojoa* when reared on the columnar cactus species utilized by its derived relatives. The well-assembled and annotated *D. navojoa* genome will be instrumental in identifying genes involved in this host shift.

*Drosophila navojoa*, *D. arizonae*, and *D. mojavensis* form a triad with known divergence times and distances (Sanchez-Flores et al. 2016). The degrees of hybrid incompatibility, that is, hybrid sterility and inviability, have been well documented (Ruiz et al. 1990). Lopez-Maestre et al. (2017) were able to identify the misexpression patterns in hybrids between *D. arizonae* and *D. mojavensis.* These 2 species diverged from each other some 1.5 million years ago (mya). Although the divergence between these 2 and *D. navojoa* was more than 5 mya, they still hybridize, although less successfully (Ruiz et al. 1990). Examining expression patterns in the hybrids between more distant relatives can help distinguish early versus late appearing genetic incompatibilities during evolution. It will be also interesting to study the evolutionary rates of protein-coding genes across more recent time scales, as presented by Guillén et al. (2018) for the more divergent *D. buzzatii* and *D. mojavensis* (this issue).

Finally, and of great interest, is the role of chromosomal inversions in speciation in the *mojavensis* cluster. Examining the sequences at inversions breakpoints is only possible if a species’ genome has a first-rate assembly, as demonstrated by Delprat et al. (2018) for the *D. mojavensis* chromosomes (this issue). Several inversions separate *D. mojavensis* and *D. arizonae* from each other and from *D. navojoa* (Ruiz et al. 1990). With reliable assemblies for the 3 species, the sequences at and adjacent to these breakpoints and the evolutionary forces underlying their maintenance can be more precisely studied.

## Funding

The laboratory of ABC was supported by grants from the Wellcome Trust - UK (grant 207486/Z/17/Z), Coordenação de Aperfeiçoamento de Pessoal de Nível Superior - Brazil, Fundação Carlos Chagas Filho de Amparo à Pesquisa do Estado do Rio de Janeiro - Brazil, and Conselho Nacional de Desenvolvimento Científico e Tecnológico - Brazil; NONY and the transcriptome study were supported by Consejo Nacional de Ciencia y Tecnología - Mexico (grant CB180385).

## Supplementary Material

Supplementary Figure 1

Supplementary Material

## Data Availability

The improved genome assembly of *D. navojoa* genome, along with the transcriptome assembly and the new annotation were deposited in NCBI under the accession number LSRL00000000.
