# Supplementary material for: An Improved Genome Assembly for Drosophila navojoa, the Basal Species in the mojavensis Cluster
Source: J Hered. 2018 Nov 13;110(1):118–23. doi: 10.1093/jhered/esy059 (PMC6321958; doi:10.1093/jhered/esy059)

Vanderlinde_SupMat_Figure1, An improved genome assembly for *Drosophila navojoa*, the basal species in the *mojavensi*s cluster, JHered

Supplemental Figure 1. Contaminants in the *D. navojoa* genome assembly. Blobplot of the initial *D. navojoa* assembly identifying contamination with bacterial genome. Each scaffold is plotted based on its GC content (x axis) and coverage (y axis), with a diameter proportional to its length and colored by its assignment to phylum. The histograms above and to the right of the main plot sum contig spans for GC proportion bins and coverage bins, respectively.

./nav4_refseq.blobDB.json.bestsum.phylum.p7.span.500.blobplot.spades

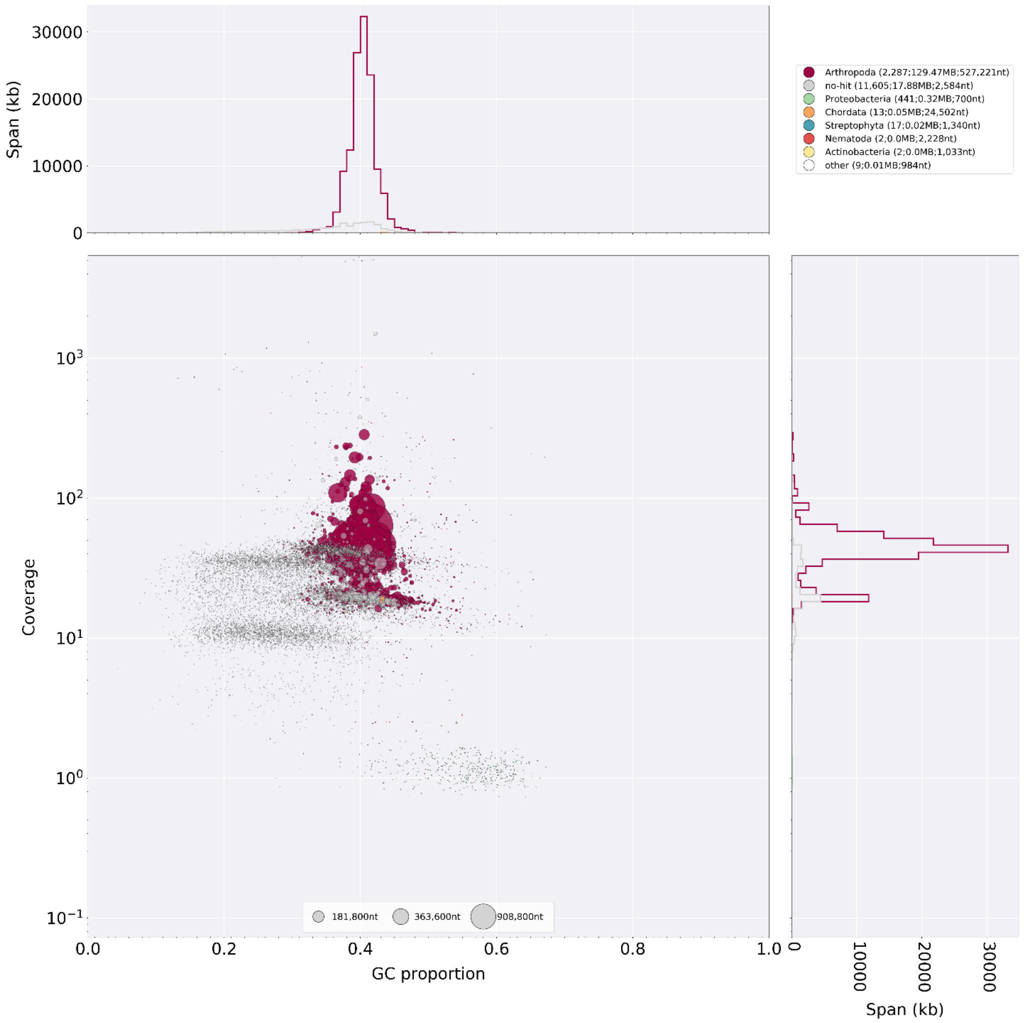

Supplement: Supplementary Figure 1 [file esy059_suppl_supplementary_figure.pdf]
