# Supplementary material for: An Improved Genome Assembly for Drosophila navojoa, the Basal Species in the mojavensis Cluster
Source: J Hered. 2018 Nov 13;110(1):118–23. doi: 10.1093/jhered/esy059 (PMC6321958; doi:10.1093/jhered/esy059)
Supplement: Supplementary Material [file esy059_suppl_supplementary_material.pdf]

Vanderlinde_SupMat_Command_lines, An improved genome assembly for *Drosophila navojoa*, the basal species in the mojavensis cluster, JHered

Software version and command lines used in all bioinformatics analyses

Genomic data

Flash (version 2.2.0)

```
nohup flash2 -z  -t 2 SRR7958874_1 SRR7958874_2 &
```

SPAdes (version 3.11.1)

```
nohup spades.py -o nav4 -m 800 -t 40 \
--pe1-1 SRR7958874_1 --pe1-2 SRR7958874_2 \
--mp1-1 SRR7958875_1 --mp1-2 SRR7958875_2 &
```

Busco (version 3.0.2)

```
nohup python3 /home/tools/busco/scripts/run_BUSCO.py --in nav4.fas --out busco_nav4 \
--lineage_path /home/tools/busco/diptera_odb9 --mode genome --long -c 8 -sp fly
>nav4_long.nohup &
```

Blobtools (version 1.0)

```
# First, preparing the hits-file (check https://blobtools.readme.io/ for details)
# RefSeq_protein database downloaded from ftp://ftp.ncbi.nlm.nih.gov/blast/db/
# prot.accession2taxid.gz downloaded from ftp://ftp.ncbi.nlm.nih.gov/pub/
nohup diamond blastx --block-size 2 --query nav4.fasta --max-target-seqs 100 --
sensitive --index-chunks 1 --threads 32 -d /draft1/blast_db/refseq_protein.dmnd \
 --evalue 1e-25 --outfmt 6 qseqid staxids bitscore sseqid sstart send qstart qend --
taxonmap /draft1/blast_db/prot.accession2taxid.gz --out nav4_refseq.taxa.m8 &
# Blobtools
blobtools create -i nav4.fasta -t nav4_refseq.taxa.m8.sedit -y spades -o nav4_refseq
blobtools view -i nav4_refseq.blobDB.json -o ./
blobtools plot -i nav4_refseq.blobDB.json --noreads -l 500 -o ./
# Removing low-coverage contaminant reads
# Creating a list with scaffolds with coverage up to 4x
awk '/NODE/{print $0}' nav4_refseq.blobDB.table.txt | awk '($2>=500)' | awk '( $5>4
){print $1}' | sed 's/_/ /g' | awk '{print $2}' >nav4_filtered_list.txt
# extracted valid scaffolds with blastdbcmd (blast+)
```

```
makeblastdb -dbtype nucl -in nav4.fas -out nav4

blastdbcmd -db nav4 -entry_batch nav4_filtered_list.txt >nav4_filtered.fas
```

Augustus (version 3.3.1)

```
# Preparing the hintsfile:

nohup blat -minIdentity=92 nav4_filtered.fas nav4_mRNA_spades.fasta
nav4_blat_mRNAspades.psl &

nohup pslCDnaFilter -maxAligns=1 nav4_blat_mRNAspades.psl nav4_blat_mRNAspades.f.psl &

nohup blat2hints.pl --in= nav4_blat_mRNAspades.f.psl --out=
nav4_blat_mRNAspades.hints.E.gff &

# augustus command, BUSCO_nav4_1181434100 is the name of the metaparameters directory
created by BUSCO.

nohup augustus --species=BUSCO_nav4_1181434100 --hintsfile=
nav4_blat_mRNAspades.hints.E.gff --extrinsicCfgFile=extrinsic.ME.cfg nav4_filtered.fas
>nav4_busco_mRNAspades.gtf &
```

CD-HIT (version 4.7)

```
# We clustering the Drosophila navojoa predicted proteins with proteins from 6
Drosophila species (D. melanogaster, D. pseudoobscura, D. willistoni, D. mojavensis, D.
virilism, D. grimshawi) from FlyBase.org.

# All these proteins are concatenated in the file Drosophila_ptns_concatenated.fas

nohup cd-hit -i Drosophila_ptns_concatenated.fas -c 0.8 -M 64000 -T 32 -o
Dro6_nav4.2_clusters.cdhit &
```

Transcriptome data

Trinity (version 2.5.1)

```
nohup Trinity --seqType fq --max_memory 60G \
 --left SRR7973855_1,SRR7973856_1,SRR7973857_1,SRR7973858_1 \
 --right SRR7973855_2,SRR7973856_2,SRR7973857_2,SRR7973858_2 \
 --CPU 5 --no_bowtie --min_kmer_cov 2 --output nav_transcriptome &
```

SPAdes (version 3.11.1)

```
nohup spades.py -m 500 -t 16 --rna \
--pe1-1 SRR7973855_1 --pe1-2 SRR7973855_2 \
--pe1-1 SRR7973856_1 --pe1-2 SRR7973856_2 \
--pe1-1 SRR7973857_1 --pe1-2 SRR7973857_2 \
--pe1-1 SRR7973858_1 --pe1-2 SRR7973858_2 \
-o nav_transcriptome &
```

Busco (version 3.0.2)

```
nohup python2.7 /home/tools/busco/scripts/run_BUSCO.py \
```

```
-in nav_transcriptome.fas --out BUSCO_nav_transcriptome \
--lineage_path /home/tools/busco/diptera_odb9 --mode transcriptome &
```
